# Developing a text-message library for tobacco prevention among adolescents: A qualitative study

**DOI:** 10.1371/journal.pone.0296503

**Published:** 2024-01-04

**Authors:** Georges Elias Khalil, David McLean, Erica Ramirez, Paris Piere Mihaj, Bairu Zhao, Biswadeep Dhar, Meerah Khan

**Affiliations:** Department of Health Outcomes and Biomedical Informatics, University of Florida, Gainesville, Florida, United States of America; University of Leeds, UNITED KINGDOM

## Abstract

**Introduction:**

Communicating the risks associated with nicotine and tobacco use to adolescents can be challenging, especially with the current tobacco market’s attempt to capture the attention of youths. Text message interventions have emerged to address the need to improve tobacco risk communication. This article informs the design of a message library for tobacco risk communication that is based on the transtheoretical model and addresses the risk of multiple tobacco products.

**Methods:**

We draw findings from this study from two phases. Phase 1 involved six remote focus group discussions (n = 25) and an in-depth interview, and Phase 2 involved online ideation sessions (n = 11) that led to the current version of the messages. We conducted the study within a larger project for the design and testing of a tobacco prevention program. With thematic analysis and the affinity mapping technique, two research team members identified repeated topics and relevant quotes to organize them into themes and subthemes.

**Results:**

In Phase 1, thematic analysis revealed four major themes: 1) Adolescents’ gap in tobacco knowledge, 2) Social influence and popularity, 3) Attitude toward marketing, and 4) Text message framing preferences. During Phase 2, participants generated 1-to-7 iterations of the original messages. Votings and discussions resulted in a library of 306 messages under 7 sections, categorized based on the processes of change from the transtheoretical model.

**Conclusion:**

The current study presents key insights crucial for developing and evaluating a library of tobacco prevention text messages that is scientifically valid and successfully resonates with today’s adolescents. Our future plan is to go beyond this initial message development and vet the message library by adolescents and expert reviewers in tobacco risk communication. Future research may consider developing messages that are tailored based on gender, ethnicity, and other factors that are predictive of tobacco use.

## Introduction

### Background

Tobacco use remains a leading cause of mortality in the United States, and nearly all tobacco users initiate during adolescence [1]. Communicating the risks associated with nicotine and tobacco use to adolescents can be challenging, especially with the current tobacco market’s attempt to capture the attention of youths. Advertisement to youth by the tobacco industry has been predominantly at retail stores and online through carefully crafted marketing campaigns. These campaigns tend to focus on the aesthetics of products, including their thin appealing shape and their availability in a variety of flavors [2–5]. There is ample evidence that adolescents’ exposure to the marketing of any tobacco product is associated with their tobacco initiation by the time they reach young adulthood [6]. Even adolescents’ exposure to non-cigarette marketing has been found to predict the initiation of cigarette smoking [6]. Given the continuously changing tobacco market, successful innovative strategies to communicate the risk of tobacco use to this vulnerable population are crucial.

Text message interventions have emerged to address both the need to improve tobacco risk communication and reach a wider audience [7]. One key benefit of health text messages is their versatile application. For instance, they can be combined with existing interventions, disseminated through mobile health programs, or even embedded in social media campaigns. A second benefit of health text messages is their relatively low cost in implementation and the delivery of content uniformly across a population [7]. Several text message interventions have been developed for adolescents, aiming to improve a variety of health outcomes, such as weight management [8], physical activity [9], mental health [10], diabetes outcomes [11], and social well-being [8, 12].

Several researchers have developed and tested text-message interventions for youth smoking cessation but with varying results. A meta-analysis by Mason and colleagues indicated that the efficacy of these interventions is not uniform [7]. Part of this inconsistency stems from differences in the text message content and framing styles. In addition, studies varied in the number of delivered text messages, and a dose-response relationship was identified between the number of text messages being delivered and the effect size of the intervention. Hence, It is important to develop a large library of text messages that is uniform in its content and style. Additionally, about one-third of the studies that were reviewed did not report a theoretical foundation for the text messages. This highlights the need for further research on the development of text messages for tobacco prevention and cessation among adolescents.

Recent preliminary research has begun to develop text messages for adolescents, covering multiple forms of tobacco products (i.e., combustible tobacco and vaping products). The results of a study on secondhand smoke and secondhand vaping indicated that half of the participating adolescents reduced their cotinine levels as a result of a text message intervention [13]. Another pre-post study developed and tested text messages for vaping risk communication [14]. The results indicated improvement in adolescents’ knowledge about the harms of vaping and their perception of vaping risk. While the design of a comprehensive form of text messaging is relatively recent, it shows promising results and encourages the development of a text-message library that covers multiple forms of tobacco products.

### Theoretical framework

The theoretical foundation is key to the success of behavioral interventions, particularly those that aim to change health behaviors long-term. One theoretical framework that has been widely applied for tobacco prevention and cessation is the transtheoretical model of behavior change (TTM) [15, 16]. According to the TTM, individuals move through stages of change until they maintain healthy behavior. The processes of change are cognitive, affective, and evaluative mechanisms that allow individuals to transition from one stage to another. Examples of such processes include consciousness-raising (i.e., understanding the facts), counterconditioning (i.e., using substitutes for unhealthy behavior), and reinforcement management (i.e., using rewards for healthy behaviors). In the context of a text messaging intervention, the processes of change not only suggest solutions for behavior change but also serve as tailored strategies based on one’s stage of change, thereby contributing to a personalized intervention.

### Study setting

Developing text messages in partnership with adolescents from after-school organizations can be particularly beneficial. Previous research suggests that after-school organizations work to promote youth empowerment [17], reduce substance use [18–21], and improve the general quality of life [22]. As a result, working closely with after-school organizations can allow the involvement of highly motivated and proactive adolescents during the design of messages for tobacco prevention.

### Study aim and strategy

This article informs the design of a message library for tobacco risk communication that is based on the TTM and addresses the risk of multiple tobacco products. Particularly, we aim to: (1) determine contextual and social features of tobacco use, knowledge, and beliefs that might facilitate the identification of content needed for the development of health messages and (2) design the message database through a participatory approach.

## Materials and methods

### Study design

The study was conducted within a project that aimed to design and test a tobacco prevention intervention for adolescents. The findings from this qualitative study were drawn from two phases. Following the development of an initial set of messages, we conducted qualitative research under Phase 1 through remote focus group discussions (n = 25) and an in-depth interview to inform key information for message refinement. We finalized with remote co-design ideation sessions under Phase 2 that led to the current version of the messages. Remote qualitative sessions have the advantage of being conducted in a synchronous format and with geographically diverse participants. They also allow adolescents to participate from the comfort of their homes. The main procedures for Phase 1 and Phase 2 of the study are described in the S1 Table.

### Inclusion/exclusion criteria

The study was open to English-speaking adolescents between the ages of 11 and 18 who participated in after-school programs. Adolescents had to have access to a webcam, a computer, and the internet to participate online owing to COVID-19 restrictions that hindered the staging of in-person sessions.

### Recruitment and sampling strategy

We recruited participants for this study between August 4, 2020, and October 27, 2020. For Phase 1, we recruited participants from two youth organizations in Northern and Central Florida (the 4-H Program and the Boys and Girls Clubs) and a registry of potential research participants from underserved Florida counties through HealthStreet, a local community engagement program. HealthStreet conducts community outreach to connect community members with available health resources and opportunities, including research participation [23, 24]. During Phase 1, we first identified 37 parents of eligible adolescents and then approached them over the phone or via video conferencing. Of 28 interested parents, 27 had adolescents who were also interested and participated in the study. Two participants were randomly selected to participate in a paired in-depth interview, and 25 adolescents participated in six focus group discussions with 4 to 7 participants per group. Two participants dropped out halfway through the session, and we kept the data that they provided up to the time they left. For Phase 2 (i.e., the message design sessions), 40 adolescents shared an interest in being part of the youth design committee (YDC), and 11 of them participated in the message design sessions. None of the participants in Phase 2 were participants in Phase 1. We had an average of 5.57 participants per month, with 3 to 7 participants per session. The research team had access to information that could identify individual participants during data collection and the qualitative sessions.

### Ethics statement

This article adheres to the consolidated criteria for reporting qualitative research (COREQ; S2 Table). The study was reviewed and approved by the University of Florida Institutional Review Board (IRB). Parents of adolescents signed informed written consent forms, and adolescents completed an informed written assent form online [25]. A research coordinator informed them of the study’s purpose and procedure. Participants were instructed to be in a room alone for a private session in a safe and relaxed environment. The moderator asked participants to maintain the confidentiality of their identity and the identity of others during the session. The moderator allowed participants to take breaks and told participants that they could withdraw from the study at any time. Phase 1 sessions were recorded using Zoom, and we made backup recordings. For Phase 1, after a session, participants received a $10 gift card each as compensation. For Phase 2, the online sessions were not recorded, data was collected in writing, and participants in the YDC received compensation with a total of up to $75.

### Developing the first version of the library

For Phase 1, before the qualitative sessions, we first developed 148 messages using the processes of change from the TTM [26]. First, we conducted a content analysis of a comprehensive evidence-based program for tobacco prevention called ASPIRE [27], to identify all the tobacco-related topics that need to be covered for each process of change. Then, the research team developed several messages covering each topic, to end up with an initial library.

The messages covered ten processes of change, including consciousness-raising, dramatic relief, environmental reevaluation, social liberation, self-reevaluation, self-liberation, stimulus control, counterconditioning, reinforcement management, and helping relationships. We also included two key topics of discussion in adolescent tobacco prevention programs: reinforcement of beliefs and advocacy [28]. Table 1 presents a description of the processes of change and examples from the first version of these messages.

**Table 1:** Processes of change and message examples.

Processes of Change	Definition	Message Examples from The Initial Version
Consciousness-raising	Enhancing understanding of oneself and the issue at hand	Tobacco products contain Polonium-210, which is a nuclear waste, just one of the things tobacco companies try to hide from us.
Dramatic relief	Expressing emotional reactions to the health challenges and potential solutions	Nothing is as gross as a never-ending cough. Tobacco does this to you.
Environmental reevaluation	Examining how the health issue affects the physical and living environment	There are over 7,000 chemicals in tobacco smoke, even after a smoker breathes out.
Social liberation	Increasing awareness of alternative behaviors that are healthy in society	Parents easily tell if their kid is using tobacco. The smell never goes away, because you cannot wash your lungs!
Self-reevaluation	Assessing personal feelings and thoughts with regard to the health issue	We may think we look cool when vaping, smoking, or dipping, but the way tobacco messes with our looks is crazy, making us look old without us realizing.
Self-liberation	Making a firm commitment to engage in a healthy behavior	Today is the day to make a final decision. We can commit to a tobacco-free life.
Stimulus control	Managing stimuli that can promote unhealthy behaviors	The easiest way to refuse tobacco is to simply say no, politely. "No thanks" is straightforward, and works most of the time.
Counterconditioning	Replacing unhealthy behaviors with healthier alternatives	Talking to a counselor can be helpful when our friends and family aren’t.
Reinforcement management	Rewarding self when engaging in healthy behaviors	When life is getting overwhelming, it can help to take a break. Powering through leads to burnout, and that doesn’t help anyone.
Helping relationships	Being open to receiving others’ support	Feeling down? Reach out to a friend. Talking about your problems can be very therapeutic.
Reinforcement of beliefs	Strengthening personal beliefs about healthy behaviors	You can share the truth on social media! Telling your friends and followers what the tobacco companies are doing, and how tobacco is hurting us is a great way to spread the word.
Advocacy	Engaging in the community to advocate for healthy behaviors	A great way to be involved in the fight against tobacco is to join an organization working to help people our age and others know the truth about tobacco.

### Study instruments

For Phase 1, a qualitative instrument was created and pilot-tested with two adolescents and two young-adult interns and then revised for completion. The instrument consisted of 41 open-ended questions that asked participants about (1) their current knowledge about tobacco product content (2) the perceived harm of tobacco use, (3) social exposure to tobacco use, and (4) ways to improve the initial library of text messages. Phase 1 aimed to support the design of the text messages by using adolescents’ conversational styles during the qualitative sessions. Following the qualitative session, participants completed a survey on their demographic characteristics, tobacco use status (i.e., cigarettes, cigars, hookah, and vaping), and susceptibility to use tobacco [29]. For Phase 2, the ideation sessions instrument included an online cloud-based table that was accessed by all the attendees of the session at the same time, allowing them to go over the initial version of the messages and improve these messages on the editable table. Details of the instruments for Phases 1 and 2 can be found under the S1 File.

### Data collection and procedures

#### Phase 1 procedures

During Phase 1, the qualitative sessions were conducted using the online video conferencing software Zoom. Qualitative procedures involved one lead researcher (GEK, gender: male, credentials: M.P.H., Ph.D.), one graduate research assistant (DM, gender: male, credentials: M.A.), and one research coordinator (ER, gender: female, credentials: B.S.). As the moderator, ER was trained in qualitative research methods and engaged in 5 rehearsal sessions using this study’s qualitative instrument. For quality assurance, a qualitative research scientist was present during the sessions and did not identify any interviewer-related biases. The moderator instructed participants to be in a room by themselves during a session and began each session with an icebreaker to help establish a relationship between participants. The icebreaker was followed by a debriefing about the session and the qualitative instrument. For quantitative data collection, participants received an online survey that was distributed via Redcap [30]. Sessions took approximately 90 minutes.

#### Phase 2 procedures

Our study team and the YDC participated in one one-hour co-design online ideation session per month for seven months, during which attendees (1) brainstormed new versions of the messages from the initial library to end up with a final library and (2) participated in the design and ideation of an interactive tobacco prevention program. The current paper only describes the results of message design. First, the moderator read each text message out loud and asked all youth committee members to brainstorm in silence, ways to reframe the message based on their preferences. With several versions written by all committee members, the moderator read every new version of the message out loud and allowed participants to discuss the new version. This was done while considering the results of the qualitative sessions at Phase 1. Participants then made additional updates to their messages and discussed the new versions until a consensus was reached for their preferred version, making a final decision on each message. During the workshop, as attendees discussed the results of the Phase 1 qualitative sessions, they were allowed to make a decision to remove a particular topic from the library or add new topics that they find important.

### Qualitative analysis

The goals for this study were to (1) determine contextual and social features of tobacco use, knowledge, and beliefs that might facilitate the identification of content needed for the development of health messages and (2) design the message database through a participatory approach. The session audio files were transcribed by a third-party transcription company. Two members of the study team went over the transcripts and identified emerging themes. New themes were captured using the grounded theory approach. Each transcript was examined to identify recurring themes and pertinent quotes. The themes changed as new information was discovered along the process, up until the point of thematic saturation. The two coders regularly met to discuss differing issues and come to a consensus. Participants who belonged to the youth design committee (YDC) were asked to provide feedback on the themes identified by the thematic analysis. Microsoft Word and Excel were used alongside Nvivo to manage the transcripts and conduct thematic analysis. In addition, one coder engaged separately in affinity mapping to categorize the quotes into themes in an agile environment. Affinity mapping is a strategic phase of the design process that allows us to extract insights and identify additional themes by visually organizing participant quotes. Also known as affinity diagramming or the Kawakita-Jiro (K-J) Method, affinity mapping is a technique for thematic analysis that allows a team to engage in an inductive and collaborative process to synthesize qualitative data into thematic groups without using predetermined categories [31]. Affinity mapping has been used successfully in previous research to efficiently group qualitative data without preexisting parameters [32].

## Results

For the qualitative sessions of Phase 1, participants (n = 25) were 65.38% female (mean age = 15.03 years, SD = 2.02). In this phase, 15 (60.00%) participants self-identified as non-Hispanic White and 12 participants (57.14%) reported being susceptible to using tobacco in the future. From our sample, 4 participants (19.05%) indicated having tried vaping products and 2 participants (8.00%) reported having tried a combustible tobacco product (cigarettes, cigars, little cigars, or hookah).

The qualitative data scripts will be made available upon request. Thematic analysis for Phase 1 revealed four major themes: 1) Adolescents’ gap in tobacco knowledge, 2) Social influence and popularity, 3) Attitude toward marketing, and 4) Preferences in text message framing. We report the results for these themes in the context of (1) participants’ general experience with tobacco and (2) their reactions to the initial version of the text messages.

### Theme 1: Adolescents’ gap in tobacco knowledge

#### Assuming a level of knowledge

The extent of adolescents’ knowledge of tobacco was revealed when participants were asked what they knew about different tobacco products. Participants were aware that cigarettes, smokeless tobacco, and cigars are dangerous in a general sense but had little knowledge of other products. Participants knew that cigarettes cause cancer, lung disease, and heart disease. They were also aware that cigarettes are highly addictive, and that quitting can be difficult. Participants did not report any knowledge of the psychological and social dangers of tobacco.

- “They often tell us that it causes like cancer and like lung cancer, and that I think it causes heart disease and some other–like blood pressure problems, and things like that” (Participant 3, Focus group 1)
- “I know cancer and heart problems and–it’s addicting. So it’s hard to quit” (Participant 1, Focus group 1)
- “It can also have–like, it affects your lungs. So it’ll make it harder to breathe and make it more difficult. It also can make you immunocompromised later in periods of time.” (Participant 1, Focus group 4)

During the focus group discussions, it became evident that adolescents possessed some awareness of the harmful components present in tobacco products, particularly cigarettes and cigars. Participants said that others their age try to avoid these products. They expressed their concerns regarding these substances, emphasizing that they should never find their way into the human body. Notably, they specifically mentioned alarming elements like metals and chemicals commonly employed as rat poison. Some adolescents were also aware of tobacco use consequences. Without evidence of knowledge, they expressed familiarity with e-cigarettes, because of the products’ popularity among their peers.

- “Like we kind of look at it like, ‘Eww, you smoke cigarettes.’ You know what I mean?… Like I see mostly older people smoking blacks and cigars and stuff. But I don’t think our age group really–because that’s really frowned upon. Like it’s kind of a double standard, but it’s really frowned upon.” (Participant 2; In-depth interview)
- “Because they’re [vapes] the most talked about and–probably the most talked about in this generation.” (Participant 3; Focus group 2)
- “I know that there’s rat poison products in cigarettes.” (Participant 5; Focus group 2)
- “Yeah [I know something about this product], you could inhale toxic metals from it.” (Participant 4, Focus group 5)

#### Unexpected tobacco information

When presented with the first version of the text messages, participants were “shocked” to learn that tobacco products contained many dangerous ingredients. Participants had strong reactions when they were told about the dangerous content of tobacco and thought messages on this topic were effective.

- “I was most shocked about the carbon monoxide. Because… you can literally die because it’s so toxic to us. So the fact that they would put it in a product and that it is legal to sell that really shocking to me” (Participant 1; Focus group 1)
- “Because when I hear like about the poisons, the toxic chemicals the effects and stuff, and it’s just like I would not want to do that” (Participant 5; Focus group 2)

While they were well-informed about cigarettes, cigars, e-cigarettes, and smokeless tobacco, they knew little about Hookah. Hookah was the most common response when participants were asked which product they knew the least about. Participants were especially surprised to learn how much smoke users inhaled when using Hookah.

- “I think it’s kind of crazy, especially with the–the pipe one. Like 100 cigarettes… if you think about it, that’s a lot of packs.” (Participant 5; Focus group 2)
- “[I know the least about the hookah] because I never like–even though I’ve seen it, I’ve never really understood hookah.” (Participant 1; Focus group 3)

#### A need for new information

Adolescents expressed that they have been exposed to anti-tobacco messages for much of their life and said that they were tired of repeatedly being told the same thing. Some messages covered things participants already knew, and these messages were disliked because they were “Obvious” (Participant 1; Focus group 1) or “Common sense” (Participant 2; In-depth interview) and were not needed. One participant thought messages containing information they knew were “Boring” and hearing them repeated was “*Annoying and Obnoxious*” (Participant 4; Focus group 4).

- “So just it gets old… we don’t want to hear it anymore” (Participant 3; Focus group 4)
- “And then there’s no safe tobacco product, I mean, we hear that all the time, again, with the like–sort of like statements that are just like–we all know” (Participant 1; Focus group 4)

### Theme 2: Social influence and popularity

#### Social exposure

Adolescents mentioned the prevalent nature of tobacco use within social contexts, particularly for vaping. Many adolescents shared their experiences of being exposed to tobacco, both through their peers and even within their own homes during family events or gatherings. This exposure highlighted the pervasive presence of tobacco in their social circles.

- “People who come to parties, they’ll be using them… Family members.” (Participant 3; Focus group 1)
- “Since they see that their friends are doing it, they like kind of become more open to it.” (Participant 2; In-depth interview)
- “It could be any party, yeah. It’s likely there’s going to be some type of smoking.” (Participant 1; Focus group 2)
- “That’s the hookah. I know there’s like hookah lounges in Gainesville. Like I know before Covid, like my friend, she had a birthday party at a hookah lounge downtown one time.” (Participant 2; Focus group 3).

Participants also discussed the popularity of E-cigarettes at sporting events and parties and how they can be hard to escape. Vaping is also common in schools where adolescents tend to sneak off and find places to hide and vape.

- “Like I’ve seen people go into the school bathrooms and vape.” (Participant 4; Focus group 2)
- “Like clubs and like–like parties–most definitely friend’s parties, or it could be with family and friends too. It could be any party, yeah. It’s likely there’s going to be some type of smoking.” (Participant 1; Focus group 2)
- “Like definitely at like parties and like football games and, you know, the bathroom, like at school, like the gym… But it’s just like yeah, like anywhere, anywhere they can like find a ducked off spot. Yeah, they’re going to hit that Juul.” (Participant 2; In-depth interview)

#### Social belonging and social image

Adolescents acknowledged that some of their peers use tobacco to feel a sense of belonging and inclusion within their social groups and improve their social image. They explained that they wanted to look “cool” and “fit in”.

- “Probably because they just want to do it, just because. And to show their friends like they’re cool and stuff.” (Participant 3; Focus group 5)
- “Mostly why teens get addicted to nicotine or drugs is that they don’t want to be lame in front of their friends.” (Participant 5; Focus group 5)

#### Diffused trendy products

Adolescents’ desire for social belonging and the aspiration to enhance their social image can be attributed to their exposure to a pervasive image associated with tobacco use, especially vaping. Adolescents revealed that tobacco, particularly vaping, had gained popularity and diffused as a prevalent trend or innovation within their social circles. Adolescents reported observing new and emerging tobacco products’ widespread acceptance and adoption within their peer groups.

- “It’s like seen as cool, it’s like pictured as cool in like movies and stuff, and like when you’re a young age you see it all the time in movies.” (Participant 1; Focus group 4)
- “They’re [vapes] the most talked about and–probably the most talked about in this generation.” (Participant 3; Focus group 2)
- “Everyone had Juuls, like the freshmen, sophomore, like everyone had like the Juuls.” (Participant 2; In-depth interview)

#### Peer pressure

Participants were also quick to mention the prevalence of peer pressure and how it can often lead people to start using tobacco products. They expressed that friends’ tobacco use can play a key role in influencing their decisions, showcasing the powerful impact of social influence within their circles.

- “But then like peer pressure most definitely, like what causes people to do it.” (Participant 1; Focus group 2)
- “I would say the biggest one [reason why people my age might start using tobacco] is peer pressure.” (Participant 4; Focus group 6).

During the evaluation of the initial version of text messages, it was noted that some adolescents found the messages addressing peer pressure as the most impactful. This preference stemmed from their personal experiences with peer pressure. Participants found these messages to resonate with their struggles and encounters with peer pressure. The discussions surrounding the influence of friends and the challenges of resisting tobacco use within their social circles affected them based on their personal experiences. Additionally, participants highlighted the importance of having the “right friends”, a social circle that supports them and helps them stay tobacco-free.

- “For me, it’s probably the ones that [are] about having the right friends [that] can help. That’s very important to me because I’ve seen a lot of peer pressure. I’ve experienced it myself. And I’ve come to the realization that having the right friends is very important because if you have the wrong friends, they can lead you down somewhere that you really don’t want to go.” (Participant 3; Focus group 6)

### Theme 3: Attitude toward marketing

#### Teen-tailored marketing

When asked why people their age use tobacco, participants highlighted that several tobacco products are targeted toward youth, particularly those that are electronic and include flavors. Participants mentioned this concern when presented with text messages regarding tobacco marketing. They were particularly surprised that marketing of tobacco products tends to be tailored to a wide variety of customer types, including youth.

- “… vapes and stuff is like more catered towards the younger age. Because it’s electronic and there were flavors at one point. And so that was sort of like catering to our age.” (Participant 1; In-depth interview)
- “I thought they usually targeted certain groups. But it sounds–if they’re marketed to everybody, that was pretty shocking to me.” (Participant 1; Focus group 1)
- “… it’s like seen as cool, it’s like pictured as cool in like movies and stuff, and like when you’re a young age you see it all the time in movies.” (Participant 1; In-depth interview)

#### Product appeal

The effect of tobacco marketing is accumulated by the product appeal, particularly with vaping products. Flavors are a key appealing characteristic of vaping products, and participants find this appeal to be a strong motivator of youth vaping.

- “… another thing is it comes in like fruity flavors, which is very appealing to people.” (Participant 4; Focus group 2)
- “Some have flavors which make a lot of teens get attracted to it.” (Participant 5; Focus group 5)

#### Misinformation

Participants reported that tobacco advertisements can be misleading, particularly when it comes to the marketing of vaping products. They explained that tobacco companies would not be looking after their customers if they misled them. When presented with the first version of the text message, participants expressed surprise to learn that tobacco companies that sell cigarettes can also sell vaping products, advertising them as a healthier choice, but they are not surprised to learn that not surprised that they would mislabel their products when they involve harmful content.

- “Misinformation. Some people think that vaping isn’t all that harmful because it isn’t cigarettes.” (Participant 1; Focus group 6)
- “… how with the companies that are creating like the cigarettes and then selling you e-cigs, like saying that they’re like less addictive.” (Participant 3; Focus group 4)
- “I feel like they’re kind of not really looking out for the people that use their products, because they’re giving false information or they’re saying whatever they have to say to make people think that their product isn’t as dangerous as it really is.” (Participant 1; Focus group 1)
- “[I am] not surprised that these companies are willing to put these dangerous products out on the market. I’m not surprised that the labels would be like false. (Participant 1; Focus group 1)

### Theme 4: Preferences in text message framing

#### Preference for empathetic messages

When discussing the anti-tobacco message used in the game, adolescents disliked messages that they saw as heavy-handed. They advised taking a softer tone to not offend people who use tobacco. Participants were worried that there could be a backlash if messages sounded judgmental.

- “Because if it becomes too strong an opinion, I think it would make those that may have already been involved with tobacco products see it in–it would be tough for them to absorb the facts, because they’d become more defensive about it.” (Participant 2; Focus group 4)
- “I think a lot of them [messages] would be received as judgmental. Because I know people get addicted to things for other underlying reasons. And I think if someone heard someone saying this about them, it would only make their situation worse.” (Participant 4; Focus group 6)
- “[Some messages are] portraying it in a very negative sense, which I feel like… you’ve got to be a little bit more lighthearted with things.” (Participant 1; Focus group 4)

Participants were also concerned about the tone of messages discussing some of the health outcomes of tobacco use. Adolescents felt that messages focusing on mental health were too negative and may offend or put off people who have mental illnesses.

- “I feel like this is one of them, because people who have like anger management issues and depression and anxiety and stuff like that, sort of have like an insecurity about that. And they, you know, can’t really control that as well as other people.” (Participant 1; Focus Group 4)

Some participants suggested that testimonial messages can help convey one’s experience with tobacco with fewer judgments.

- “… maybe show like actual people that use tobacco and like how it affected–let them talk about how it affected their life. Because it’s more like convincing if someone’s actually there telling you what they went through.” (Participant 1; Focus group 1)

#### Low self-efficacy versus confidence

By keeping in mind tobacco users’ lost self-efficacy, some participants highlighted the importance of designing messages that are sensitive to the challenges associated with quitting. They emphasized the need for mindful design, considering the difficulty that tobacco users may face during the quitting process. Conversely, when presented with motivational messages, participants expressed the belief that improving self-efficacy by empowering individuals to maintain a tobacco-free lifestyle can be helpful.

- “… because like some people may get offended or feel uncomfortable about [me talking to them about tobacco] because often people want to quit, but they can’t. [You could] make someone feel like uncomfortable if you approach them about it. (Participant 1; Focus group 1)
- “I think that’s really important to realize that just because you started don’t mean that you have to continue, and that you have the power to be able to fight back.” (Participant 3; Focus group 6)
- “My [favorite messages were] the ones where talks about the cheer, the power to basically overcome these habits. A lot of people think that once they start that they can’t stop, that they need to live off of smoking. But if they really think about it, and they really think that we can get out of this situation, we have the power to get out of the troubles. And to figure out what causes us to get in these troubles and staying away from these problems to try to keep ourselves out.” (Participant 4; Focus group 6)

#### Messages on appearance and aesthetics

Participants noted that messages regarding physical attractiveness were effective. Messages that highlighted how tobacco use would change the way they looked were impactful, in their opinion, particularly when the message allowed them to picture their change in appearance due to tobacco use.

- “It’s a lot more about like the–what it physically does to you, and I know a lot of people care about their appearance. So this may be really important to them, about what they look like.” (Participant 1; Focus group 1)
- “… the descriptiveness of it [losing teeth], where I can almost imagine like what you’re talking about. Like… it kind of makes me like think about what it would look like, and it kind of grosses you out.” (Participant 2; In-depth interview)

One message that mentioned acne was singled out for its effectiveness and relevance to adolescents who are already dealing with acne.

- “I feel like everyone would feel the first one, about the acne. Because we’re already going through enough acne on our own. I don’t want anything else to be contributing to that.” (Participant 2; In-depth interview)

#### Messages that convey statistics

Adolescents reported that they find factual messages, especially those that convey statistical data, effective. Many felt facts, especially numbers, can help adolescents realize the severity of the harm. Some reported that the messages can be more interactive by showing data for different scenarios (e.g., taking healthy and unhealthy actions).

- “I think the one with the numbers, because people like to see numbers to know like, like, I think tend to attract people more. Like I know I kind of scanned and saw the 200 and it popped out at me.” (Participant 1; Focus group 1)
- “It’s because once people would see numbers, like they’ll know like okay, this will really do some harm to my body due to the percentage that like it has with it. (Participant 3; Focus group 1)
- “I feel like it should’ve been more interactive, like here is what will happen but here’s how you can have healthy coping mechanisms instead of turning to this. And understand why it’s not good and why it’s better to do something else.” (Participant 4; Focus group 2
- “Maybe show how addicting it is. Like possibly show like how many times you use it. Like how many times smoking a cigarette will cause you to become addicted. (Participant 1; Focus group 1)

#### Updated library of text messages (Phase 2)

For Phase 2, of those who participated in the YDC (n = 11), 9 (81.82%) were female (mean age = 16.06 years, SD = 1.65). For Phase 2, nine YDC members (81.82%) identified as non-Hispanic White, and 3 members (27.27%) were susceptible to using tobacco in the future. During the design sessions, participants brainstormed between 1 and 7 iterations of the initial version of the messages. Following discussions and consensus, the design sessions resulted in a library of 306 messages under 7 sections, categorized based on the processes of change from the TTM. Table 2 presents examples of messages before and after design.

**Table 2:** Examples of messages before and after design.

Processes of change	Messages from the initial version	Messages from the new version
Consciousness-raising	Hookahs are exotic instruments that give a smooth smoke, even while giving the experience of smoking about 100 cigarettes in every session.	Hookah looks like a water vase with a pipe. While you may not use it regularly, you end up inhaling 100 cigarettes worth of tobacco in each session.
Dramatic relief	Nicotine overstimulates the region of the brain responsible for desire, pleasure, and reward, making the person less able to control what they do and how much they use tobacco.	Nicotine overstimulates the part of the brain responsible for desire, pleasure, and reward, making us less able to control what we do and how much we use tobacco
Environmental reevaluation	By smoking, you can even lose your house! In 2011, firefighters had to attend to over 90,000 calls about smoke related fires.	Your decision to smoke could cause you to lose your house! In 2011, firefighters responded to over 90,000 calls about smoke related fires.
Social liberation	Your friends can see it too. Ruining a good time and putting them in danger with secondhand smoke is not such a good idea.	Chances are, your friends do not enjoy tobacco smoke ruining a good time or putting their health in danger.
Self-reevaluation; Self-liberation	If we avoid tobacco, whether smoking, vaping, or dipping, we would not get the chance to create vapor tricks or taste different artificial flavors of fruits, or show off something new to friends, or hang on to something when we stress or get angry, but is all this worth it?	While vape tricks and cool flavors sound like fun, they’re not worth the long term health problems.
Stimulus control; Counterconditioning; Reinforcement management; Helping relationships	Get involved in activities you enjoy. When you feel sad or down, this can feel annoying. But it will be worth it. Choose something you’ve enjoyed in the past, whether it be a sport, an art, dance, music, reading, or writing	It might be hard, but going out and doing the things you love can help you feel more like yourself.
Helping relationships; Reinforcement of beliefs; Advocacy	People who smoke tobacco often know the consequences but just can’t seem to stop. offering emotional support can be the last push they need	People who smoke tobacco often know the consequences but just can’t seem to stop. We can be the support they need to finally quit.

Our library of messages presented topics that are common for tobacco education under each process of change from the TTM. In addition, the library included topics that are unique to new and emerging products (vaping products and hookah), and it considered sub-themes that we identified during the qualitative sessions conducted in Phase 1. Each group of messages covered an average of 4.28 sub-themes. We also retrieved 238 key terms that researchers and public health professionals can use to identify certain messages of interest (Table 3).

**Table 3:** Library of messages by topics and covered keywords and subthemes.

Processes of change	Covered topics	Subthemes covered from Phase 1	Additional message keywords
Consciousness-raising (40 messages)	What is tobacco; Content of tobacco products; Content that is also in vapes and hookah	Assuming a level of knowledge; Unexpected tobacco information; A need for new information; Product appeal; Messages that convey statistics	Vaping products; Nicotine; Chemicals; Shapes and sizes; Cigars; Hookah; Tobacco products; Polonium-210; Poisonous chemicals; Arsenic; Acetone; Carbon monoxide; Formaldehyde; Succinic Acid; Lead; Ammonia; Benzene; Safer choice; Flavors; Addiction; Nicotine content; Toxic chemicals; Vitamin E; Toxins; Cancer; Vape pens; E-juice; Nicotine poisoning; Water vapor; Nicotine-free; nicotine overdose; Risks of vaping; Smoking hookah; Nicotine addiction; Hot coals
Dramatic relief (81 messages)	Tobacco and your body; Tobacco and the brain; Tobacco and your health	Assuming a level of knowledge; Unexpected tobacco information; A need for new information; Messages on appearance and aesthetics; Messages that convey statistics	Toxic chemicals; Hair; Fingers; Gum; Teeth; Cancer; Tongue; Lungs; Fungus; Skin; Voice; Pain; Nerves; Nicotine; Brain response; Attention; Learning; Neurological effects; Life expectancy; Heart; Stomach
Environmental reevaluation (36 messages)	Secondhand smoke and vapor; Thirdhand smoke and vapor; Environmental consequences	Assuming a level of knowledge; Unexpected tobacco information; A need for new information; Messages that convey statistics	First-hand smoke; First-hand vapor; Lung tissue; Cancer; Nicotine; Toxic chemicals; Flavored water vapor; Vape clouds; Label; Secondhand smoke; Secondhand vapor; Secondhand fumes; Second-hand nicotine; Disorders; ADHD; Mother’s womb; Serious diseases; Lung cancer; Stroke; Non-smokers; Asthma; Heart attack; Bar; Lung cancer; Thirdhand smoke; Aerosol; Clothing; Shoes; Furniture; Stains; Car; Dangerous chemicals; Residue; Concentrated chemicals; Kids; Accidental e-juice poisoning; Environment; Plastic can-holders; Tobacco litter; Cigarette filters; Cellulose acetate; Forest fires; Biodegradable; Recyclable; Family; Friends; Pets
Social liberation (18 messages)	How others see you if you use tobacco	Social exposure; Social belonging and social image; Diffused trendy products; Peer pressure; Low self-efficacy versus confidence	Friends; Teens; Vaping cessation programs; Quitting; Tobacco; Smoking; Harm; Perception; Cool; Sexy; Fun; Addiction; Tool; Dorks; Disapprove; Turnoff; School; Smoke breaks; Graduation; Parents; Smell; Health; Significant other; Dirty; Angry; Self-care; Tobacco-free
Self-reevaluation; Self-liberation (24 messages)	Is tobacco really what I want?; Smoking and vaping are costly; Rewards for not using tobacco and Commit to a tobacco-free life	Messages that convey statistics; A need for new information; Preference for empathetic messages	Benefits; Tobacco use; Vape tricks; Cool flavors; Long-term health problems; Looks; Slow deterioration; Brain; Calming; Addiction; Anger; Irritability; Depression; Strokes; Memory loss; Quitting; E-cigs; Replacement addiction; Smell; Clothes; Skin; Hair; Cravings; Friends; Dip; Chew tobacco; Spit; Stress relief; Cancer; Heart disease; Cost; Testimony; Health care costs; Time; Immune system; Second-hand smoke; Vapor; School suspension; Longevity; Healthier lives; Memory; Brain health
Stimulus control; Counterconditioning; Reinforcement management; Helping relationships (78 messages)	Tobacco triggers; You have the power to deal with triggers; How to deal with depression; How to deal with stress; How to deal with peer pressure; How to create healthy habits; How to Receive social support	Social belonging and social image; Peer pressure; Low self-efficacy versus confidence; Preference for empathetic messages	Triggers; Feeling down; Doubting yourself; Stressful school and work; Stress relief; Brain effects; Addiction; Cravings; Peer pressure; Fit in; Boredom; Curiosity; Smell; Popular vape flavors; Habits; Short-term gratification; Happiness; Physical and mental health; Guidance counselor; Healthcare providers; Therapists Hotlines; Support groups; Exercise; Diet; Sleep; Healthy foods; Brain exercise; Rash decisions; Support from friends; Asking for help; Online resources; New friends; Being a good listener; Negative influences; Counseling; Separating from negative influences; Reaching out; Positive influences
Helping relationships; Reinforcement of beliefs; Advocacy (29 messages)	Importance of spreading a tobacco-free message; Skills to communicate against tobacco; Advocacy in the community; Activism in the community; Activities in a group	Diffused trendy products; Teen-tailored marketing; Misinformation; Preference for empathetic messages; Low self-efficacy versus confidence	Tobacco companies; Hiding information; Trick; Safe to use; Court-ordered; Advertising; Addiction; Voices being heard; Petition; Anti-tobacco organizations; Social media; Influencers; Vape shops; Flavored e-cigs; Petitions; Campaign; Referral features; Quitting resources; Confronting friends; Supportive and encouraging; Saying no; Holding people accountable; Honest communication; Building trust; Quit plans; Face-to-face conversation; Starting a conversation; Support for quitting

## Discussion

The current study presents key insights crucial for developing and evaluating a library of tobacco prevention text messages that is scientifically valid and successfully resonates with today’s adolescents. To convey the risk of tobacco to youth, the FDA and the NIH encourage the development of effective tobacco education approaches [33]. To this end, we have taken the opportunity to develop a library of messages by applying a human-centered participatory approach. In the first phase of this study, a series of focus groups revealed key themes that informed the design of our text messages. Adolescents expressed their perception of harm, the detrimental effect of social influence and peer pressure, their attitudes toward tobacco marketing, and message design recommendations. The second phase featured these themes, facilitating adolescents’ active contribution to the development of a text message library for future tobacco prevention and education efforts. The study highlights the importance of our messages to cover adolescents’ gaps in understanding tobacco products, moving beyond risk communication. Negative social influence, including peer pressure, peer modeling, and exposure to tobacco through various channels, pose barriers to a tobacco-free lifestyle. Participants were surprised by targeted tobacco marketing toward youth, suggesting the importance of introducing anti-marketing messages. Finally, adolescents recommended a softer tone for our messages, emphasizing the importance of empathizing with tobacco users to promote a tobacco-free lifestyle. This aligns with prior research on the effectiveness of empathetic and supportive messages in inducing health-promoting responses and reducing resistance [34, 35].

First, our findings showed that adolescents had some understanding of tobacco, its content, and its consequences. While a lack of tobacco-related knowledge has been previously established (e.g. [36]), some subthemes are unique to the current study, including a misconception that they have enough knowledge, surprise when exposed to new information, and expression of the need for novel information. Although adolescents recognized the harmful outcomes of tobacco use, including nicotine vaping, they were still surprised to learn about the many physical and mental risks associated with tobacco use, such as lung injury, cancer, and nicotine dependence. In addition, adolescents highlighted some of the hurtful content of tobacco products, such as toxic metals and chemicals found in rat poison. These results align with the consciousness-raising process of the transtheoretical model, suggesting adolescents were beyond the precontemplation phase [15, 16]. As a result, while it is tobacco risk communication is important, we should go beyond and raise awareness about other relevant tobacco topics.

Corroborating previous research [37], we identified negative social influence and peer pressure as key barriers to adolescents maintaining a tobacco-free lifestyle. While social influence has been established by previous research by describing the roles of peer pressure and social belonging (fitting in with friends to avoid social exclusion) [38], other aspects of social influence (social exposure, social image, and diffusion of trendy products) are unique outcomes of the current study. Within their social circles, adolescents tend to encounter situations where they are exposed to tobacco, whether through their peers or parents, both at home and during social gatherings. On one hand, participants explained that some adolescents use tobacco to enhance their social image. On the other hand, mere exposure to tobacco users creates an expectation of tobacco use among peers, making this behavior to be perceived as a trend. Unlike tobacco use to improve a social image, certain adolescents feel pressured to use tobacco so they can maintain their friendships. Furthermore, some participants mentioned being exposed to tobacco use by celebrities, which is consistent with prior research on media influence. In line with the social learning theory [39], adolescents expressed that these social exposures contribute to the formation of negative social norms. On the other hand, it is worth noting that several participants emphasized the importance of peer support. They indicated that having friends who support a tobacco-free lifestyle can alleviate the pressure to engage in tobacco use, in line with recent research on positive influence [40]. These results suggest that health messages need to convey the complex processes of social influence to adolescents and train adolescents to recognize these processes, thereby managing them in their daily lives. Future research should also investigate the role of peer support as a potential strategy to combat negative influence and prevent tobacco use.

While participants were presented with a wide variety of messages, they were particularly enthusiastic to express their negative attitudes toward tobacco marketing. They expected that tobacco companies would take advantage of the products’ appealing characteristics such as flavors and smell, but they were surprised to learn that marketing is tailored toward several age groups, including adolescents. They also explained that while it may be counterintuitive for tobacco companies to mislead their customers, this may be expected, considering the dangers that come out of tobacco use. This research aligns with prior qualitative research that calls for increased regulation of social media messaging and marketing of tobacco, with a focus on marketing that appeals to adolescents [41]. In designing our text messages, we made sure to address the appealing characteristics of tobacco products, particularly vaping products, debunk misconceptions, and address the harmful effects of tobacco use. Future research could explore the application of our text messages as anti-marketing messages for adolescents, creating a strategy to counteract tobacco marketing. Investigating adolescents’ reactions to anti-marketing messages can provide insights into how we can improve this strategy for a comprehensive campaign.

During Phase 1, to improve our messages, adolescents recommended that we use a softer tone to not offend tobacco users. They believed there could be resistance from tobacco users if messages sounded judgmental. With that in mind, we ensured that the YDC played a key role in designing judgment-free messages that are empathetic and supportive. Previous research has shown that empathy can induce cognitive and emotional processes that reduce counterarguing, prevent resistance, and ultimately evoke health-promoting responses [42, 43]. In support of such messages, it is key to improve adolescents’ self-efficacy and allow the messages to motivate them to engage in healthy behaviors. As a result, the current library of text messages can promote self-efficacy by presenting adolescents with motivational messages and tangible “How to” messages that support stimulus control, counterconditioning, and reinforcement management. One way to boost self-efficacy is to introduce an interactive system through an ecological momentary intervention that would allow adolescents to reply to these messages and share their engagement in healthy behaviors to maintain a tobacco-free lifestyle.

The results of our qualitative work led to a comprehensive text message library, with 306 messages under the processes of change from the TTM, covering key topics of tobacco that allow adolescents to understand tobacco content and its risks, reevaluate their decision regarding tobacco, learn how to maintain a tobacco-free lifestyle, and advocate against tobacco in their communities. Our future plan is to progress on the message development and vet the message library by expert reviewers in risk communication and tobacco control, and adolescents. Reviewers can rate each message for its persuasive capacity. The aim is to confirm the agreement between the reviewers, the researchers, and the YDC.

### Strengths and limitations

In this study, we designed a message database through a participatory approach, considering contextual and social characteristics of tobacco use, knowledge, and beliefs among adolescents. Through our qualitative approach, we uncovered key individual and social factors that adolescents consider to be key to designing successful health messages. Although the text message library can be further improved, this library is deemed ready for testing among adolescents. Our work highlighted the design needs during Phase 1 and applied the findings to the design process with adolescents’ participation during Phase 2. This is one of the first studies to identify key messaging features within a comprehensive library of messages for tobacco risk communication, including ways for adolescents to take action through positive conversations with their peers and advocacy against tobacco use. The findings highlighted the need for these message topics to be further developed during future studies.

As a limitation, although there were more female than male participants over the two phases, the study’s findings offered qualitative findings from both genders equally. In addition, we distributed the two genders at random across groups, thereby ensuring equal gender representation within groups. Also, the two phases had distinctly different demographics. While Phase 1 included a racially and ethnically diverse sample, Phase 2 was conducted with a sample that was predominantly non-Hispanic White. While Phase 2 involved participants who were randomly selected from Phase 1, non-Hispanic White participants from Phase 1 may have been more likely to accept participating in Phase 2, compared to participants who identify with other ethnic groups. As opposed to random sampling, an intentionally selected group with similar demographic characteristics to the greater teenage population may have been a more appropriate choice for Phase 2. Despite the demographic differences between participants from the two phases, the results showed that the YDC from Phase 2 approved the majority of the qualitative reports and covered the sub-themes obtained from Phase 1, as they were designing the messages. While the current messages were not designed to be tailored to particular ethnic groups, future research may consider developing tailored messages that consider gender and ethnicity. Considering that the messages were designed based on the transtheoretical model of change, they can be applied in a tailored fashion based on an adolescent’s stage of behavior change. In addition, adolescents may receive different messages that fall under different topics of interest based on adolescents’ needs with respect to tobacco information (themes can be found in Table 3). After identifying an adolescent’s stage of change through ecological momentary assessment, a tobacco risk communication campaign can then deploy a set of messages based on the adolescent’s stage.

Although a larger number of participants during Phase 1 may have resulted in a more representative sample, a focus group study with 15 participants is typical in qualitative research. According to earlier qualitative research, we can achieve thematic saturation with 15 adolescents. In addition, the use of a relatively small homogeneous group of participants in Phase 2 of the design process is common practice during participatory design [44]. Phase 2 participants were predominantly at low risk of tobacco use. This was expected, considering that adolescents with the most motivation to volunteer in the design of these programs are already at a lower risk of tobacco initiation. However, Phase 2 participants were proactively in contact with adolescents who are at high risk of tobacco use, and they engaged in the design process based on their experience with their at-risk peers. It is possible that adolescents’ tobacco knowledge may have influenced their qualitative reports and message design. While we did not quantitatively assess tobacco knowledge in this study, we extensively explored adolescents’ level of knowledge during the Phase 1 focus groups, and participants in Phase 2 acknowledged the results obtained during Phase 1. Furthermore, the YDC was joined by the research team and a tobacco education scientist who validated the message content during the ideation sessions.

### Implications

To advance research about adolescent tobacco prevention via text messages, we plan to engage in additional key areas of study. Firstly, message evaluation studies are needed to examine the impact of the text message library on adolescents’ knowledge, attitudes, and behaviors related to tobacco use. These studies put ideas into action in real-world contexts, receiving input from experts, researchers, and adolescents to assess their persuasive power and involvement. Second, by disseminating the messages through a text messaging campaign, it is possible to conduct a longitudinal study that can provide important information about the long-term impact of the text-message intervention among adolescents. With such a study, researchers can also determine any relapse or diminishing effects by following participants over time. Furthermore, it is critical to investigate the impact of peer support as a method for combating negative social influence and preventing tobacco use. Researchers can develop and test peer support interventions that allow adolescents to exchange and diffuse anti-tobacco text messages (e.g., through social media), to see if they can encourage healthy behaviors, reshape social norms, and prevent tobacco use initiation. Ultimately, this line of research can help to further build and improve the text message library, and it contributes to the growing body of research supporting the application of text messages as a successful tool for teenage tobacco prevention. The results of these studies can have practical implications for future public health initiatives aimed at reducing teenage tobacco use.

## Supporting information

S1 File Study instruments. (PDF)

S1 Table Main procedures for Phase 1 and Phase 2 of the study. (PDF)

S2 Table COREQ checklist. (PDF)
