# Supplementary material for: Developing a text-message library for tobacco prevention among adolescents: A qualitative study
Source: PLoS One. 2024 Jan 4;19(1):e0296503. doi: 10.1371/journal.pone.0296503 (PMC10766181; doi:10.1371/journal.pone.0296503)
Supplement: S1 File — (PDF) [file pone.0296503.s001.pdf]

Focus Group Instrument
Text Messages

Phase 1

Introduction

- **Disclaimers**
  - All opinions are valuable
  - Everything said is confidential
  - Please raise your hand when you have something to say
  - Any questions?
- **Introduction to topics discussed in Focus Groups**
  - Answer questions to help create a program
  - Any questions?
- **Brief Overview of Zoom**
  - What to do if you get disconnected from zoom
    - check your email for contact information to help get reconnected
    - any questions?
- **Introducing members**
  - Introduce ourselves by saying our name and one fun fact about ourselves

Introduction

-“Hi everyone! Thank you for joining us today for this group discussion! Before we start, I would like to go over a few important points.”
-As we engage in this group discussion, please remember that all opinions are very valuable to us; there are no right or wrong answers, we just want to learn about your thoughts, so please don’t be afraid to speak up! Although, you are not required to answer questions that make you uncomfortable.
-Everything you say or type in Zoom meeting is confidential. Your parents, teachers, or others at your program will not know what was said in this meeting. Only research staff will have access to this information.
-Please raise your hand if you have feedback so we can make sure everyone has a turn to talk.
-Do you have any questions so far?

Intro to topics discussed in focus group
-In this group discussion, you will answer questions that will help us design a new board game. We will mostly ask questions that will help us improve the content.
-Does anyone have any questions about that?

Brief overview of Zoom 30sec
What to do if you get disconnected from Zoom
Do you have any questions so far?

Introduction of members in focus group 50sec
To get to know each other better, let's introduce ourselves by saying your name and one fun fact about yourself. **For example, interests, hobbies, favorite things…**

Survey

- It's time to take a survey!

- Ok everyone, Before we begin the group discussion, I would like everyone to work on the survey. My colleague will send a link to the survey in the Zoom chat once I go over a few things.
  - We will complete this survey now, and will have another quick survey to complete at the end of the group discussion.
  - This survey may ask you questions about tobacco as well as a few questions about yourself. If you're having a hard time answering the questions, please ask me for help. I'm more than happy to help you understand what the question is asking!
  - Your answers to this survey will be kept confidential. Only our research staff will have access to your answers, so please be as honest as possible when answering the questions.
  - You are not required to answer any questions that make you feel uncomfortable.
  - There are no right or wrong answers to the questions in this survey.
  - The survey should take about 10 minutes to complete.
  - At the end of the survey, please press submit.
- My colleague has sent out the link to the survey in the private chat on Zoom. Did you receive the link?
  - [have them confirm receiving survey]
  - [if they didn't receive the link, moderator will send again]

- Please let me know if you need any help with the survey! Once everyone is done, we can begin the group discussion.
- Ok, let's move on with the group discussion!

Tobacco Products

- I am going to show you pictures of tobacco products. For each product can you tell me what you know about the product (what they're called, how they're used, etc.).
- Do you have any questions before I start?

Can you tell me what you know about these products?

[Cigarettes Image]

Tobacco Products

Can you tell me what you know about these products?

[Images of vaping products]

Tobacco Products

Can you tell me what you know about these products?

[Images of cigars, little cigars, and cigarillos]

Tobacco Products

Can you tell me what you know about these products?

[Image of hookah]

Tobacco Products

Can you tell me what you know about these products?

[Images of smokeless and chewing tobacco]

Tobacco Products

- Which ones do you know most about?
- Which ones do you know least about?
- What would you like to know about these products?

Cigarette

- tube-shaped paper packed with tobacco leaves
- Lit on one end and inhale smoke into the lungs
- Contains nicotine and other chemicals

Cigar

- tube-shaped tobacco wrapper tightly packed with tobacco leaves
- lit on one end and smoke is not inhaled into the lungs
- Contains nicotine and other chemicals

Dip/chew

- Type of tobacco product that is not smoked or burned
- May be used as chewing tobacco or “inhaled” through the nose
- Contains nicotine and other chemicals

Electronic Cigarette (e-cig)

- Product that uses battery and does not contain tobacco
- Contains nicotine and other chemicals
- Nicotine turns into a mist and is inhaled directly into the lungs

Hookah/shisha/water pipe

- Vase-shaped tobacco product that uses charcoal to heat up the tobacco
- Contains nicotine and other chemicals
- The product has water for the heated tobacco to pass through the tube and the combination is inhaled

Tobacco Questions

- Which of these tobacco products do people your age like most?
- What are reasons people your age might start using tobacco?
- What are reasons people your age might start vaping?
- What do people your age like the least about these products?
    - Are there tobacco products that you know of that we did not mention?

Tobacco Questions

- What have you learned about tobacco in school/health programs?
  - How good were these programs?
  - What were some of the things they talked about?

Tobacco Questions

- In your opinion and based on what you know, what are the harms of using tobacco that you know about?
    - What are the harms of vaping?

Tobacco Questions

- In what type of settings or social gatherings have you seen tobacco being used?
  - What kind of people were at the social gathering?

- How would you talk to your friends/family about their tobacco use?
  - When your friends or family members talk about tobacco, what are some things they say?
  - What are some ways you can help the community to fight tobacco use?
  - How do they usually get these products?

Environment

- What do you think of when you hear the word environment?
- What do you and your family do to help the environment?
- What are the changes that you have seen happen in your lifetime that were made to help the environment?
    - What do you think about these changes?
- If we were to compare tobacco and its effects to a big storm, what would that look like?

Messages

We’re going to go over some information and ask you your opinion on them.

Questions:

- What is your first reaction to the messages in this section?
- What parts of the message appeal to you the most?
- What parts of the message do you find to be not so convincing?
- How would you edit the message to make it better?
- What would you add or remove in this message to make it better?
- [Messages from the first version, on the following topics]

- What are tobacco products?
- Content of Tobacco Products
- Content Is Also in Vapes and Hookah
- External Physical Effects of Tobacco Products
- Brain Effects of Tobacco and Addiction
- Smoking and Vaping are Costly
- Medical Consequences of Tobacco Use
- How Others See You If You Use Tobacco
- Tobacco Triggers
- Receive social support
- Second/third-hand smoke and vape & environmental
- Advocacy & Activism

Ideation Workshop Sessions Instrument

Phase 2

Phase 2 online instrument for the ideation sessions

Initial version of messages	YDC member 1*	YDC member 2	YDC member 3	YDC member 4	Study team member 1*	Study team member 2	Final version of the message	Processes of change and keywords
Message 1								
Message 2								
Message 3								
…								

YDC stands for youth design committee; *The number of YDC members and study team members varied per session
