# Supplementary material for: Developing a text-message library for tobacco prevention among adolescents: A qualitative study
Source: PLoS One. 2024 Jan 4;19(1):e0296503. doi: 10.1371/journal.pone.0296503 (PMC10766181; doi:10.1371/journal.pone.0296503)
Supplement: S1 Table — (PDF) [file pone.0296503.s002.pdf]

S1 Table

Table: Main procedures for Phase 1 and Phase 2 of the study

Procedures	Phase 1	Phase 2
**Objective**	Understanding of adolescents' preferences concerning tobacco prevention messages	Refinement of message content and ideation of interactive tobacco prevention messages
**Participants**	Adolescents	Adolescents
**Number of Participants**	n=25	n=11
**Setting**	Remote (online video conferencing)	Remote (online video conferencing)
**Data Collection Method**	Recording sessions	Note-taking and filling out tables in real-time
**Engagement Style**	The moderator presents open-ended questions and participants answer and interact with each other.	Co-design sessions, whereby participants and study team members interact to correct existing messages, write new ones, and make a final consensus.
**Session Outline**	Icebreaker, debriefing, and qualitative open-ended questions	Brainstorming, independent creative writing, discussion, and consensus-building on messages
**Duration**	Approximately 90 minutes	One one-hour session per month for seven months
**Facilitators/ Moderators**	GEK (lead researcher), DM (graduate research assistant), ER (research coordinator)	GEK (lead researcher), DM (graduate research assistant), ER (research coordinator)
**Analysis**	Thematic analysis with the affinity mapping technique	Content analysis and categorization of messages by process of change, health topics, and subthemes covered in Phase 1.
**Outcome**	Common themes from qualitative sessions	Final library of messages for tobacco prevention
**Support for Updating the Messages**	Participants shared attitudes and opinions regarding tobacco and tobacco messages.	Participants made direct updates based on creative writing, discussions, and consensus
**Additional Decision-Making**	None	The participants made decisions to remove or add topics based on Phase 1 results and discussions.
