# Supplementary material for: Developing a text-message library for tobacco prevention among adolescents: A qualitative study
Source: PLoS One. 2024 Jan 4;19(1):e0296503. doi: 10.1371/journal.pone.0296503 (PMC10766181; doi:10.1371/journal.pone.0296503)
Supplement: S2 Table — (PDF) [file pone.0296503.s003.pdf]

S2 Table: COREQ checklist

Items	Information	Location in manuscript
**Domain 1: Research team and reflexivity**		
**Personal Characteristics**		
1. Interviewer/facilitator: Which author/s conducted the interview or focus group?	One lead researcher (GEK, gender: male, credentials: M.P.H., Ph.D.), one graduate research assistant (DM, gender: male, credentials: M.A.), and one research coordinator (ER, gender: female, credentials: B.S.) conducted the sessions.	Materials and Methods, Data Collection
2. Credentials:<br><br>What were the researcher's credentials? E.g. PhD, MD	The researchers on this project had the following credentials: B.S., M.A., M.P.H., and Ph.D.	Materials and Methods, Data Collection, page 11, line 229
3. Occupation: What was their occupation at the time of the study?	Research Coordinator, Research Assistant, and Assistant Professor.	Materials and Methods, Data Collection
4. Gender: Was the researcher male or female?	The main researcher was male, and the main moderator was female.	Materials and Methods, Data Collection

5. Experience and training: What experience or training did the researcher have?	The moderator was trained in qualitative research methods and engaged in 5 rehearsal sessions using this study's qualitative instrument.	Materials and Methods, Data Collection
**Relationship with participants**		
6. Relationship established: Was a relationship established prior to study commencement?	Yes. A relationship was established through ice-breaker activities prior to the collection of quantitative data.	Materials and Methods, Data Collection
7. Participant knowledge of the interviewer: What did the participants know about the researcher? e.g. personal goals, reasons for doing the research	Participants were briefed on the purpose of the study and understood that it was a research project about tobacco prevention and that their feedback would be used in the development of a board game.	Materials and Methods, Ethics Statement
8. Interviewer characteristics: What characteristics were reported about the interviewer/facilitator? e.g. Bias, assumptions, reasons and interests in the research	There were no interviewer-related biases identified in this study.	Materials and Methods, Data Collection
**Domain 2: study design**		

9. Methodological orientation and Theory: What methodological orientation was stated to underpin the study? e.g. grounded theory, discourse analysis, ethnography, phenomenology, content analysis	Once participants were interviewed, members of the study team went over the transcripts and identified emerging themes. New and emerging themes, following grounded theory, were captured using open coding and thematic analysis. Each transcript was examined to identify recurring themes and pertinent quotes. The themes changed as new information was discovered along the process, up until the point of thematic saturation. One coder engaged separately in affinity mapping to categorize the quotes into themes in order to allow us to extract insights and identify additional themes by visually organizing participant quotes.	Materials and Methods, Qualitative Analysis
**Participant selection**		
10. Sampling: How were participants selected? e.g. purposive, convenience, consecutive, snowball	In Phase 1, participants were recruited from two youth organizations in Northern and Central Florida (the 4-H Program and the Boys and Girls Clubs) and a registry of potential research participants from underserved Florida counties through HealthStreet, a local community engagement program. In Phase 2, adolescent participants from Phase 1 shared interest in being part of the youth design committee (YDC), and thus were recruited conveniently.	Materials and Methods, Recruitment and Sampling Strategy

11. Method of approach: How were participants approached? e.g. face-to-face, telephone, mail, email	Parents of eligible adolescents were approached over the phone or via video-conferencing.	Materials and Methods, Recruitment and Sampling Strategy
12. Sample size: How many participants were in the study?	25	Results
13. Non-participation: How many people refused to participate or dropped out? Reasons?	Two participants dropped out during the phase 1 sessions because they were no longer interested in the content of the session.	Materials and Methods, Recruitment and Sampling Strategy

**Setting**		
14. Setting of data collection: Where was the data collected? e.g. home, clinic, workplace	The qualitative sessions were conducted using the online video conferencing software Zoom. For quantitative data collection, participants received an online survey that was distributed via Redcap. In Phase 1, data was recorded by recording using Zoom, while in Phase 2, data was collected in writing.	Materials and Methods section, Data Collection & Procedures
15. Presence of non-participants: Was anyone else present besides the participants and researchers?	No.	Materials and Methods section, Data Collection & Procedures

16. Description of sample: What are the important characteristics of the sample? e.g. demographic data, date	Phase 1 participants were 65.38% male with a mean age of 15.03 years (SD = 2.02). In Phase 2, participants were 81.82% female, and they had an average age of 16.06 years (SD = 1.65).	Results, page 13, line 279
**Data collection**		
17. Interview guide: Were questions, prompts, guides provided by the authors? Was it pilot tested?	A qualitative instrument was created and pilot-tested with two young-adult interns and two adolescents, and then revised for completion, with 41 open-ended questions.	Materials and Methods, Study Instruments, page 11, line 214
18. Repeat interviews: Were repeat interviews carried out? If yes, how many?	No.	Materials and Methods section, Data Collection & Procedures, page 5, lines X
19. Audio/visual recording: Did the research use audio or visual recording to collect the data?	In Phase 1, the qualitative sessions were audio and video recorded using the native record feature on Zoom. A backup recording was made using a third-party software. In Phase 2, the YDC workshops were audio recorded using a handheld audio-recorder.	Materials and Methods section, Data Collection & Procedures, page 8, lines 179
20. Field notes: Were field notes made during and/or after the interview or focus group?	Yes, additional field notes were made during Phase 2.	Materials and Methods, Data Collection, page 12, line 242
21. Duration: What was the duration of the interviews or focus group?	The qualitative sessions lasted approximately 90 minutes.	Materials and Methods, Data Collection, page 12, line 240

22. Data saturation: Was data saturation discussed?	Yes, coding continued until thematic saturation was reached.	Materials and Methods, Qualitative Analysis, page 13, lines 259
23. Transcripts returned: Were transcripts returned to participants for comment and/or correction?	No.	Materials and Methods, Qualitative Analysis, page 13, lines 259
**Domain 3: analysis and findings**		
**Data analysis**		
24. Number of data coders: How many data coders coded the data?	Two coders coded for both Phase 1 and 2.	Materials and Methods, Qualitative Analysis, page 13, lines 259
25. Description of the coding tree: Did authors provide a description of the coding tree?	Thematic analysis was done using the grounded theory approach. In addition, one coder engaged separately in affinity mapping to categorize the quotes into themes in an agile environment.	Materials and Methods, Qualitative Analysis, page 13, lines 259
26. Derivation of themes: Were themes identified in advance or derived from the data?	Themes were derived from the data.	Materials and Methods, Qualitative Analysis, page 13, lines 259
27. Software: What software, if applicable, was used to manage the data?	Microsoft Word, Microsoft Excel, and Nvivo.	Materials and Methods, Qualitative Analysis, page 13, lines 271

28. Participant checking: Did participants provide feedback on the findings?	Participants who belonged to the youth design committee (YDC) were asked to provide feedback on the themes identified by the thematic analysis.	Materials and Methods, Qualitative Analysis, page 13, lines 259
**Reporting**		
29. Quotations presented: Were participant quotations presented to illustrate the themes / findings? Was each quotation identified? e.g. participant number	Yes, themes were supported with direct quotes attributed to anonymous participants by gender and focus group number.	Results, page 14-27, lines 296-607
30. Data and findings consistent: Was there consistency between the data presented and the findings?	Yes	Results, page 14-27, lines 296-607
31. Clarity of major themes: Were major themes clearly presented in the findings?	Yes, major themes were presented in the Results section.	Results, Results, page 14-27, lines 296-607
32. Clarity of minor themes: Is there a description of diverse cases or discussion of minor themes?	We did not retrieve minor themes in this study.	Results, Results, page 14-27, lines 296-607
